# *Pneumocystis jirovecii* prophylaxis with trimethoprim-sulfamethoxazole and resistance in ESBL-producing Enterobacterales bloodstream infections among kidney transplant recipients

**DOI:** 10.1186/s13756-026-01799-4

**Published:** 2026-08-18

**Authors:** Elena Pérez-Nadales, Elisa Ruiz-Arabi, Daniele Riccucci, Jordi Carratalà, Mical Paul, Mario Fernández-Ruiz, Rocío Álvarez-Marín, Isabel Carmona, Esperanza Merino de Lucas, Regino José Rodríguez-Álvarez, Miruna D. David, Seema Mehta Steinke, Ligia C. Pierrotti, Isabel Machuca, Isabel Oriol, Jesus Rodríguez-Baño, Luis Martínez-Martínez, José María Aguado, Julián Torre-Cisneros, Juan José Castón, Belén Gutiérrez-Gutiérrez, INCREMENT-SOT Study Group, Núria Sabé, Rafael San Juan, Francisco López-Medrano, María Paniagua-García, Maite Ruiz Pérez de Pipaón, Noelia Balibrea-Lara, Livia Giner-Oncina, Silvia Otero-Rodriguez, Darin Ostrander, Ángela Cano, Manuel Recio-Rufián, Maristela Pinheiro Freire, Patricia Muñoz, Alessandra Mularoni, Zeliha Koçak Tufan, Julien Coussement, Benoit Pilmis, Emmanuel Roilides

**Affiliations:** 1 CIBER of Infectious Diseases (CIBERINFEC) (CB21/13/00049), Instituto de Salud Carlos III, Madrid, Spain; 2 Maimonides Biomedical Research Institute of Cordoba, Reina Sofía University Hospital (IMIBIC/HURS/UCO), University of Cordoba, Cordoba, Spain; 3 Department of Agricultural Chemistry, Soil Science and Microbiology, University of Cordoba, Cordoba, Spain; 4 Clinical Unit of Microbiology, Reina Sofía University Hospital, Cordoba, Spain; 5 Infectious Diseases Service, Department of Surgery and Medical Sciences, Reina Sofía University Hospital, University of Cordoba, Cordoba, Spain; 6 Department of Medical and Surgical Sciences, Alma Mater Studiorum, University of Bologna, Bologna, Italy; 7 Department of Infectious Diseases, Bellvitge University Hospital- IDIBELL, University of Barcelona, Barcelona, Spain; 8 Infectious Diseases Division, Rambam Health Care Campus, The Ruth and Bruce Rappaport Faculty of Medicine, Technion - Israel Institute of Technology, Haifa, Israel; 9 Unit of Infectious Diseases, Instituto de Investigación Hospital “12 de Octubre” (imas12), “12 de Octubre” University Hospital, Universidad Complutense, Madrid, Spain; 10 Clinical Unit of Infectious Diseases, Microbiology and Parasitology, Virgen del RocíoUniversity Hospital; Institute of Biomedicine of Seville, Virgen del Rocío University Hospital/CSIC/University of Seville, Seville, Spain; 11 Infectious Diseases Unit, Dr. Balmis General University Hospital, ISABIAL, Alicante, Spain; 12 Infectious Disease Unit, Cruces University Hospital, Baracaldo, Spain; 13 Microbiology Department, Queen Elizabeth Hospital Birmingham, University Hospitals NHS Foundation Trust, Birmingham, UK; 14 School of Medicine, Johns Hopkins University, Baltimore, USA; 15 Division of Infectious Diseases, David Geffen School of Medicine, UCLA Medical Center, Los Angeles, CA USA; 16 Department of Infectious Diseases, University of São Paulo School of Medicine, Hospital das Clínicas, São Paulo, Brazil; 17 Clinical Unit of Infectious Diseases and Microbiology, D, University Hospital VirgenMacarena, Seville, Spain; 18 Department of Medicine and Microbiology, Institute of Biomedicine of Seville, University of Seville, Instituteof Biomedicine of Seville (University Hospital Virgen Macarena/CSIC/University of Seville), Seville, Spain; 19 Infectious Diseases Unit, Hospital Universitario ReinaSofía, Cordoba, Spain; 20 Instituto Maimónides de Investigación Biomédica de Córdoba (IMIBIC), Cordoba, Spain

## Abstract

**Background:**

Kidney transplant (KT) recipients are at high risk of bloodstream infection (BSI) caused by extended-spectrum beta-lactamase-producing Enterobacterales (ESBL-E). Trimethoprim-sulfamethoxazole (TMP-SMX) is widely used for post-transplant prophylaxis, but its relationship with subsequent TMP-SMX resistance in this setting remains poorly defined.

**Methods:**

We conducted a retrospective multicentre cohort study of KT recipients with monomicrobial ESBL-E BSI from the INCREMENT-SOT cohort. The primary outcome was TMP-SMX resistance; the secondary outcome was co-resistance to TMP-SMX and ciprofloxacin. The association with TMP-SMX prophylaxis during the preceding 30 days was assessed using inverse probability of treatment weighting and modified Poisson regression with centre-clustered robust variance. Additionally, exploratory Classification and Regression Tree (CART) and stratified analyses were performed.

**Results:**

Among 323 KT recipients with ESBL-E BSI, 160 (49.5%) had received TMP-SMX prophylaxis within 30 days before BSI onset. Overall, TMP-SMX resistance was detected in 267/323 cases (82.7%). Resistance was more frequent among prophylaxis recipients than among non-recipients (91.3% vs. 74.2%; *P* < 0.001). Recent prophylaxis was associated with TMP-SMX resistance in the IPTW analysis (RR, 1.18; 95% CI 1.02–1.37; *P* = 0.03) and after additional adjustment for lymphopenia, chronic liver disease, and microbiological aetiology (aRR, 1.16; 95% CI 1.00–1.35; *P* = 0.05). Exploratory CART analysis identified lymphopenia as the main splitting variable among prophylaxis recipients. In patients with lymphopenia, resistance occurred in 94.4% of prophylaxis recipients and 74.5% of non-recipients, with an IPTW-adjusted RR of 1.23 (95% CI 1.00–1.51; *P* = 0.048). TMP-SMX and ciprofloxacin co-resistance occurred in 70.3% of patients and was more frequent among prophylaxis recipients than among non-recipients (75.6% vs. 65.0%; *P* = 0.04), although the association did not reach statistical significance after IPTW and multivariable adjustment (aRR, 1.11; 95% CI 0.97–1.28; *P* = 0.10).

**Conclusions:**

In KT recipients with ESBL-E BSI, recent TMP-SMX prophylaxis was associated with a higher probability that the causative isolate was resistant to TMP-SMX. Exploratory analyses identified a particularly high prevalence of resistance among prophylaxis recipients with lymphopenia. These findings should be interpreted cautiously given the observational design and the specific study population, but they may be useful for risk stratification and for anticipating limited oral treatment options in certain clinical scenarios.

**Supplementary Information:**

The online version contains supplementary material available at https://doi.org/10.1186/s13756-026-01799-4.

## Introduction

Bloodstream infection (BSI) is a common complication in solid organ transplant (SOT) recipients and an important cause of post-transplant morbidity and mortality [1]. Among these infections, extended-spectrum beta-lactamase-producing Enterobacterales (ESBL-E) are of particular concern. Reported risk factors for ESBL-E BSI in SOT recipients include immunosuppression, healthcare exposure, and previous antimicrobial use, including trimethoprim-sulfamethoxazole (TMP-SMX) in some cohorts [2].

Trimethoprim-sulfamethoxazole (TMP-SMX) is one of the most widely used antibiotics in kidney transplant (KT) recipients and remains the first-line agent for prophylaxis against *Pneumocystis jirovecii* pneumonia (PJP) during the early post-transplant period [3]. Beyond its role in PJP prevention, TMP-SMX prophylaxis has also been associated with a reduced risk of urinary tract infections in renal transplant recipients compared with alternative prophylactic strategies [4].

Despite its widespread use, limited data are available on the potential impact of TMP-SMX prophylaxis on subsequent resistance patterns, particularly in the context of multidrug-resistant Gram-negative infections such as those caused by ESBL-E. Therefore, the aim of this study was to evaluate whether recent exposure to TMP-SMX prophylaxis was associated with TMP-SMX resistance among KT recipients who developed ESBL-E bloodstream infection. In addition, we explored co-resistance between TMP-SMX and ciprofloxacin.

## Materials and methods

### Study design and population

We conducted a retrospective, multicentre cohort study involving consecutive KT recipients with confirmed bloodstream infections (BSIs) caused by ESBL-E. Patients were identified from the INCREMENT-SOT cohort (ClinicalTrials.gov identifier: NCT02852902), an international collaborative registry comprising 38 centres across 16 countries, covering the period from 2004 to 2016 [5, 6].

Patients with polymicrobial bacteraemia were excluded in accordance with the original INCREMENT-SOT cohort design, in order to maintain a homogeneous microbiological population and allow direct attribution of the resistance phenotype to the ESBL-producing Enterobacterales isolate responsible for the index BSI. Patients who had received two or more days of active antimicrobial therapy prior to blood culture sampling were also excluded. This exclusion criterion was inherited from the original cohort design and was intended to minimise the potential influence of prolonged effective therapy before culture collection on bacterial recovery and on the microbiological characteristics of the index episode.

All included patients were followed for 30 days after the index blood culture. The study protocol was approved by the Spanish Medicines and Medical Devices Agency (AEMPS, code FIB-COL-2015-01) and the Institutional Review Board of Hospital Universitario Reina Sofía (code 2907), which waived informed consent due to the observational design. Local ethics approval was obtained at each participating centre, in accordance with institutional requirements. The study is reported in accordance with the Strengthening the Reporting of Observational Studies in Epidemiology (STROBE) recommendations; the completed checklist is provided in Supplementary Table S1.

### Variables and definitions

The primary outcome was TMP-SMX resistance in the ESBL-E isolate responsible for the index BSI, irrespective of whether the patient had received recent TMP-SMX prophylaxis. The secondary outcome was co-resistance to TMP-SMX and ciprofloxacin in the same isolate.

The independent variables were assessed at the date of blood culture collection except where specified, and included demographic data, chronic comorbidities, immunosuppressive regimens, and infection severity. The following scores were used to assess comorbidity and illness burden: Pitt bacteraemia score [7], age-adjusted Charlson comorbidity index [8], McCabe classification [9].

Additional variables included acquisition setting (nosocomial vs. non-nosocomial), admission ward type (medical, surgical, emergency department or intensive care unit), source of infection according to CDC criteria [10], presence and degree of sepsis (including severe sepsis and septic shock) [11] and the causative microorganism. Nosocomial acquisition was defined as BSI onset more than 48 h after hospital admission or within 48 h after hospital discharge; all remaining episodes were classified as non-nosocomial.

Receipt of TMP-SMX prophylaxis in the 30 days prior to bacteraemia onset was specifically recorded. Because detailed information on TMP-SMX dose, frequency, cumulative duration, treatment interruptions, and recent discontinuation was not uniformly available across centres, prophylactic exposure was operationalised as a binary variable: any TMP-SMX prophylaxis within the preceding 30 days versus no prophylaxis during that period.

A 90-day post-transplantation threshold was used to distinguish early from late BSI. The first 90 days after transplantation represent a clinically relevant period characterised by more intense immunosuppression and routine administration of TMP-SMX prophylaxis in most recipients. This variable was used exclusively as a temporal descriptor of when the BSI occurred and was not considered a surrogate for cumulative TMP-SMX exposure.

We used a threshold of 1,000 lymphocytes/µL to define lymphopenia, consistent with prior literature [12]. Maintenance immunosuppression was analysed according to individual agents received because specific combination regimens were not uniformly recorded across participating centres and could not therefore be modelled separately. Dialysis in the preceding 30 days referred to receipt of renal replacement therapy during that period, whereas chronic kidney disease reflected baseline renal dysfunction/allograft impairment as recorded in the registry.

### Microbiology

Enterobacterales isolates were identified at each participating centre using standard microbiological methods. Screening for ESBL production was performed in isolates with reduced susceptibility to third-generation cephalosporins and was confirmed by CLSI- or EUCAST-recommended phenotypic tests [13]. In some centres, confirmation was additionally performed using PCR and sequencing techniques. Isolates were classified as ESBL producers if they fulfilled phenotypic or molecular criteria.

Antimicrobial susceptibility testing was carried out at the local laboratories using automated systems or disk diffusion and interpreted according to the 2015 Clinical and Laboratory Standards Institute (CLSI) breakpoints. For isolates obtained before 2015, available minimum inhibitory concentrations (MICs) were reviewed and susceptibility categories were reassigned accordingly; when MIC data were unavailable, or when the available MIC was equal to or below the former susceptibility breakpoint, isolates were considered susceptible if they had been reported as such by the local laboratory.

Active antimicrobial therapy was defined as administration of at least one agent to which the pathogen was susceptible in vitro, given at an appropriate dose and schedule [14].

### Statistical analysis

Continuous variables were summarised using medians and interquartile ranges (IQR). Comparisons between continuous variables were conducted using the Mann–Whitney U test. Categorical variables were compared using the Chi-square test or Fisher’s exact test, as appropriate. All tests were two-sided, and a *p* value ≤ 0.05 was considered statistically significant. Associations with the primary and secondary outcomes were expressed as risk ratios (RRs) with 95% confidence intervals (CIs).

Age and the age-adjusted Charlson comorbidity index were entered into regression analyses as continuous linear terms, with estimates expressed per one-year and one-point increase, respectively. Calendar year was entered as a continuous linear term in the propensity-score model. Binary variables were entered as dichotomous indicators, and microbiological aetiology was modelled categorically, with *Escherichia coli* as the reference category. More complex non-linear terms were not included in order to preserve model parsimony, given the limited number of patients with TMP-SMX-susceptible isolates.

To account for baseline differences between patients who had and had not received TMP-SMX prophylaxis, a propensity score for receipt of prophylaxis during the preceding 30 days was estimated using logistic regression. The propensity-score model included calendar year, age, sex, chronic liver disease, diabetes mellitus, chronic kidney disease, BSI occurring within 90 days after transplantation, urinary tract stenosis, dialysis during the preceding 30 days, CMV replication during the preceding 30 days, induction immunosuppressive therapy, recent acute graft rejection, and McCabe classification. These variables were selected because of their potential association with the probability of receiving TMP-SMX prophylaxis.

Stabilised inverse probability of treatment weights (IPTW) were calculated from the propensity scores and truncated at the 2.5th and 97.5th percentiles to limit the influence of extreme weights. Propensity-score overlap was assessed by examining the distributions in the two exposure groups. Covariate balance before and after weighting was evaluated using standardised mean differences, with an absolute standardised mean difference below 0.10 considered indicative of adequate balance. The distribution of the stabilised weights and the effective sample size after weighting were also assessed.

Because TMP-SMX resistance was a common outcome, associations were estimated using modified Poisson regression with a log link and robust variance rather than logistic regression. Standard errors were clustered by participating centre to account for within-centre correlation and centre-level heterogeneity.

Classification and Regression Tree (CART) analysis was retained exclusively as an exploratory, hypothesis-generating analysis to identify potentially recognisable subgroups associated with resistance and co-resistance.

Missing data were assessed for all variables considered in the analyses. Missingness was infrequent (< 5%), and no variable included in the final models had substantial missing data. Analyses were therefore conducted using complete cases for the variables included in each model, without imputation.

All statistical analyses were performed using R software. Descriptive analyses were also conducted using SPSS version 25.0, and the exploratory CART analyses were performed using Salford Predictive Modeler version 8.2.

## Results

### Baseline characteristics

A total of 323 KT recipients developed BSIs due to ESBL-E and were included in the analysis. Of these, 160 patients (49.5%) had received TMP-SMX prophylaxis within the 30 days preceding BSI onset (Table 1).

**Table 1:** Baseline characteristics of kidney transplant recipients with ESBL-producing Enterobacterales bloodstream infection, according to TMP-SMX prophylaxis status

Variables	Study cohort		
	No TMP-SMX prophylaxis (*n* = 163)	Prophylaxis with TMP-SMX (*n* = 160)	*p*
Male gender, n (%)	88 (54.0)	89 (55.6)	0.82
Age, median (IQR), years	62.00 (52.0–69.0)	58.5 (47.7–66.0)	0.04^a^
McCabe classification, n (%)			0.06
Non-fatal	129 (79.1)	120 (75.0)	
Rapidly fatal	8 (4.9)	2 (1.2)	
Ultimately fatal	26 (16.0)	38 (23.8)	
Charlson comorbidity index (age-adjusted), median (IQR)	5.0 (4.00–8.0)	5.0 (3.0–7.0)	0.24^a^
Diabetes mellitus, n (%)	83 (50.9)	78 (48.8)	0.74
Transplant-related variables			
Induction immunosuppressive therapy, n (%) Basiliximab Antithymocyte globulin	58 (35.6) 28 (17.2)	67 (41.9) 60 (37.5)	0.25 < 0.001
Maintenance immunosuppressive therapy, n (%) Mycophenolic acid Tacrolimus Sirolimus Everolimus Ciclosporin Azathioprine Corticosteroids	120 (73.6) 115 (70.6) 8 (4.9) 9 (5.5) 30 (18.4) 9 (5.5) 18 (11.0)	140 (87.5) 144 (90.0) 2 (1.2) 9 (5.6) 13 (8.1) 8 (5.0) 12 (7.5)	0.002 < 0.001 0.10 1.00 0.008 1.00 0.34
Acute graft rejection within 30 days, n (%)	8 (4.9)	24 (15.0)	0.003
Simultaneous kidney–pancreas transplant, n (%)	3 (1.8)	5 (3.1)	0.46^b^
Chronic kidney disease, n (%)	92 (56.4)	115 (71.9)	0.004
Chronic liver disease, n (%)	21 (12.9)	16 (10.0)	0.49
Dialysis within previous 30 days, n (%)	16 (9.8)	41 (25.6)	< 0.001
Urinary tract stenosis at BSI onset, n (%)	16 (9.8)	41 (25.6)	< 0.001
ICU stay within previous 30 days, n (%)	18 (11.0)	26 (16.2)	0.20
CMV replication within previous 30 days, n (%)	10 (6.1)	25 (15.6)	0.007
CMV disease within previous 30 days, n (%)	7 (4.3)	15 (9.4)	0.08^b^
Lymphocyte count at BSI onset, median (IQR), cells/µL	900 (535–1300)	561 (277.5–990.0)	< 0.001^a^
Lymphocyte count < 1000 cells/µL	98 (60.1)	126 (78.8)	< 0.001
BSI episode-related variables			
BSI within 90 days post-transplant, n (%)	24 (14.7)	114 (71.2)	< 0.001
Urinary tract source of infection, n (%)	129 (79.1)	140 (87.5)	0.05
Nosocomial acquisition, n (%)	51 (31.3)	86 (53.8)	< 0.001
Severe sepsis or septic shock, n (%)	27 (16.6)	27 (16.9)	0.94
Pitt bacteraemia score, median (IQR)	1 (0-1.5)	0 (0–1)	0.60^a^
Altered mental status at presentation, n (%)	27 (16.6)	29 (18.1)	0.71
Source control achieved, n (%)	28 (17.2)	61 (38.1)	< 0.001
Microbiology			
Enterobacterales			0.01
*Escherichia coli*, n (%)	116 (71.2)	90 (56.2)	
*Klebsiella* spp., n (%)	40 (24.5)	64 (40.0)	
*Enterobacter* spp., n (%)	3 (1.8)	3 (1.9)	
Others, n (%)	4 (2.4)	3 (1.9)	
Outcome			
TMP-SMX resistance, n (%)	121 (74.2)	146 (91.3)	< 0.001
TMP-SMX and ciprofloxacin co-resistance, n (%)	106 (65.0)	121 (75.6)	0.04

TMP-SMX, trimethoprim-sulfamethoxazole; IQR, interquartile range; CMV, cytomegalovirus; ICU, intensive care unit; BSI, bloodstream infection

*p* values were calculated by χ2 test, except where otherwise specified;

^a^ Mann-Whitney U test. ^b^ Fisher’s exact test;

The cohort’s median age was 61 years (IQR, 50–68), and the median Charlson comorbidity score was 5 (IQR, 4–8). The median interval from transplantation to BSI was 138 days (IQR, 35–1366), with 138 episodes (42.7%) occurring within the first 90 days after transplantation.

The predominant causative pathogens were *Escherichia coli* (206 cases, 63.8%) and *Klebsiella* spp. (104 cases, 32.2%). Basiliximab and antithymocyte globulin were used as induction therapy in 125 (38.7%) and 88 (27.2%) patients, respectively. The most frequently prescribed maintenance immunosuppressive agents included mycophenolic acid derivatives (80.5%) and tacrolimus (80.2%). Acute graft rejection occurred in 9.9% of patients within the 30 days before BSI.

Compared with non-prophylaxis patients, those receiving TMP-SMX prophylaxis were younger and more frequently received antithymocyte globulin, mycophenolic acid derivatives, and tacrolimus, whereas ciclosporin was less frequently used. They also had higher frequencies of recent acute graft rejection, chronic kidney disease, dialysis during the preceding 30 days, urinary tract stenosis, CMV replication, lymphopenia, nosocomial acquisition, and achievement of source control (Table 1). They also had lower lymphocyte counts at BSI onset and a higher frequency of lymphopenia (< 1000 cells/µL) (78.8% vs. 60.1%, *p* < 0.001). In addition, TMP-SMX recipients more frequently developed BSI within the first 90 days post-transplant (71.2% vs. 14.7%, *p* < 0.001), had more nosocomial episodes (53.8% vs. 31.3%, *p* < 0.001). Microbiologically, their BSIs were more often caused by *Klebsiella spp*. and less often by *E. coli* (*p* = 0.01). Severe sepsis or septic shock was similar in both groups (16.9% vs. 16.6%, *p* = 0.94).

Propensity-score distributions showed acceptable overlap between groups. After weighting, 11 of 14 covariates had absolute standardised mean differences below 0.10 (mean, 0.069; maximum, 0.235); stabilised weights ranged from 0.508 to 4.189, and the effective sample size was 192.4.

### TMP-SMX resistance

TMP-SMX resistance was identified in 267 of 323 episodes (82.7%) and was significantly more common among patients who had received prior prophylaxis (91.3%) compared with those who had not (74.2%, *p* < 0.001).

When stratified by time from transplantation, TMP-SMX resistance remained more frequent in prophylaxis recipients both within and beyond the first 90 days after transplant. Among episodes occurring within 90 days, resistance was observed in 92.1% of prophylaxis recipients and 75.0% of non-recipients (*p* = 0.01); among those occurring after 90 days, the corresponding figures were 89.1% and 74.1%, respectively (*p* = 0.03) (Table 2). Although overall resistance was lower in episodes occurring beyond 90 days than in early post-transplant episodes (77.8% vs. 89.1%), it remained high in both strata.

**Table 2:** Resistance to TMP-SMX and co-resistance to TMP-SMX plus ciprofloxacin stratified by time from transplant to bloodstream infection, according to TMP-SMX prophylaxis in the previous 30 days

Time from transplant	Overall cohort (*n*/*N*, %)	TMP-SMX prophylaxis within 30 days prior to BSI	No TMP-SMX prophylaxis within 30 days prior to BSI	*p*-value
TMP-SMX resistance				
≤ 90 days	123/138 (89.1)	105/114 (92.1)	18/24 (75.0)	0.01
> 90 days	144/185 (77.8)	41/46 (89.1)	103/139 (74.1)	0.03
TMP-SMX plus ciprofloxacin co-resistance				
≤ 90 days	100/138 (72.5)	86/114 (75.4)	14/24 (58.3)	0.09
> 90 days	127/185 (68.6)	35/46 (76.1)	92/139 (66.2)	0.20

TMP-SMX, trimethoprim-sulfamethoxazole; BSI, bloodstream infection

In the unadjusted analysis, recent TMP-SMX prophylaxis was associated with a 23% higher probability of TMP-SMX resistance (RR, 1.23; 95% CI 1.11–1.36; *P* < 0.001). After IPTW adjustment and use of centre-clustered robust variance, the association was attenuated but remained statistically significant (RR, 1.18; 95% CI 1.02–1.37; *P* = 0.03). Following additional adjustment for lymphopenia, chronic liver disease, and microbiological aetiology, recent prophylaxis remained associated with a higher probability of resistance, although the estimate was of borderline statistical significance (aRR, 1.16; 95% CI 1.00–1.35; *P* = 0.05). In the same IPTW-weighted multivariable modified Poisson model, chronic liver disease was independently associated with TMP-SMX resistance (aRR, 1.18; 95% CI 1.04–1.34; *P* = 0.01) (Table 3 and Supplementary Table S2).

**Table 3:** Risk ratios for TMP-SMX resistance according to prior TMP-SMX prophylaxis

Model	RR for TMP-SMX prophylaxis	95% CI	*P* value
Unadjusted	1.23	1.11–1.36	< 0.001
IPTW with centre-clustered robust variance	1.18	1.02–1.37	0.03
IPTW with centre-clustered robust variance and additional adjustment for lymphopenia, chronic liver disease, and aetiology*	1.16	1.00–1.35	0.05

*See Supplementary Table S2

The exploratory CART analysis identified lymphopenia as the main splitting variable among patients who had received TMP-SMX prophylaxis (Fig. S1). In this group, TMP-SMX resistance was observed in 94.4% of patients with lymphopenia, compared with 79.4% of those without lymphopenia. Among patients who had not received prophylaxis, lymphopenia was not selected as a relevant classifier; instead, *K. pneumoniae* aetiology was the principal splitting variable, with resistance observed in 87.5% of patients with *K. pneumoniae* BSI and in 69.9% of those with other aetiologies. These CART findings were considered exploratory and hypothesis-generating.

On the basis of the pattern suggested by CART, we subsequently examined the association between recent TMP-SMX prophylaxis and resistance according to lymphopenia status. Among patients without lymphopenia, resistance was observed in 48 of 65 patients without prophylaxis (73.8%) and in 27 of 34 patients with prophylaxis (79.4%); the IPTW-adjusted association was not statistically significant (RR, 0.97; 95% CI 0.68–1.37; *P* = 0.84). Among patients with lymphopenia, resistance was observed in 73 of 98 patients without prophylaxis (74.5%) and in 119 of 126 patients with prophylaxis (94.4%), corresponding to an IPTW-adjusted RR of 1.23 (95% CI 1.00–1.51; *P* = 0.048) (Supplementary Table S3).

### Co-resistance to TMP-SMX and ciprofloxacin

Overall, co-resistance to TMP-SMX and ciprofloxacin was observed in 227 of 323 episodes (70.3%). It was more frequent among patients with recent TMP-SMX prophylaxis than among those without prophylaxis (121/160 [75.6%] versus 106/163 [65.0%]; *P* = 0.04).

When stratified by time from transplantation, co-resistance remained numerically more frequent among prophylaxis recipients both within and beyond 90 days after transplant, although these differences did not reach statistical significance (75.4% vs. 58.3%, *p* = 0.09 for episodes within 90 days; 76.1% vs. 66.2%, *p* = 0.20 for episodes beyond 90 days) (Table 2).

Recent TMP-SMX prophylaxis was associated with co-resistance in the unadjusted analysis (RR, 1.16; 95% CI 1.01–1.34; *P* = 0.04). However, this association was attenuated and was no longer statistically significant after IPTW adjustment with centre-clustered robust variance (RR, 1.13; 95% CI 0.98–1.29; *P* = 0.07) or after additional adjustment for lymphopenia, chronic liver disease, and microbiological aetiology (aRR, 1.11; 95% CI 0.97–1.28; *P* = 0.10). In the same fully adjusted model, chronic liver disease showed a borderline association with TMP-SMX and ciprofloxacin co-resistance (aRR, 1.17; 95% CI 0.99–1.38; *P* = 0.06) (Supplementary Tables S4 and S5).

The exploratory CART analysis identified a similar descriptive pattern for TMP-SMX and ciprofloxacin co-resistance (Fig. S2). Among patients who had received TMP-SMX prophylaxis, lymphopenia was the main splitting variable, with co-resistance observed in 79.4% of patients with lymphopenia and in 61.8% of those without lymphopenia. Among patients who had not received prophylaxis, *K. pneumoniae* aetiology was the principal classifier, with co-resistance observed in 77.5% of patients with *K. pneumoniae* BSI and in 61.0% of those with other aetiologies. These CART findings were considered exploratory and hypothesis-generating.

On the basis of the pattern suggested by CART, we subsequently examined the association between recent TMP-SMX prophylaxis and co-resistance according to lymphopenia status. Among patients without lymphopenia, co-resistance was observed in 43 of 65 patients without prophylaxis (66.2%) and in 21 of 34 patients with prophylaxis (61.8%); no adjusted association was observed between prophylaxis and co-resistance (RR, 0.99; 95% CI 0.67–1.46; *P* = 0.96). Among patients with lymphopenia, co-resistance was observed in 63 of 98 patients without prophylaxis (64.3%) and in 100 of 126 patients with prophylaxis (79.4%), corresponding to an IPTW-adjusted RR of 1.28 (95% CI 0.96–1.70; *P* = 0.08)(Supplementary Table S6).

### Sensitivity analyses

**Fig. 1:**
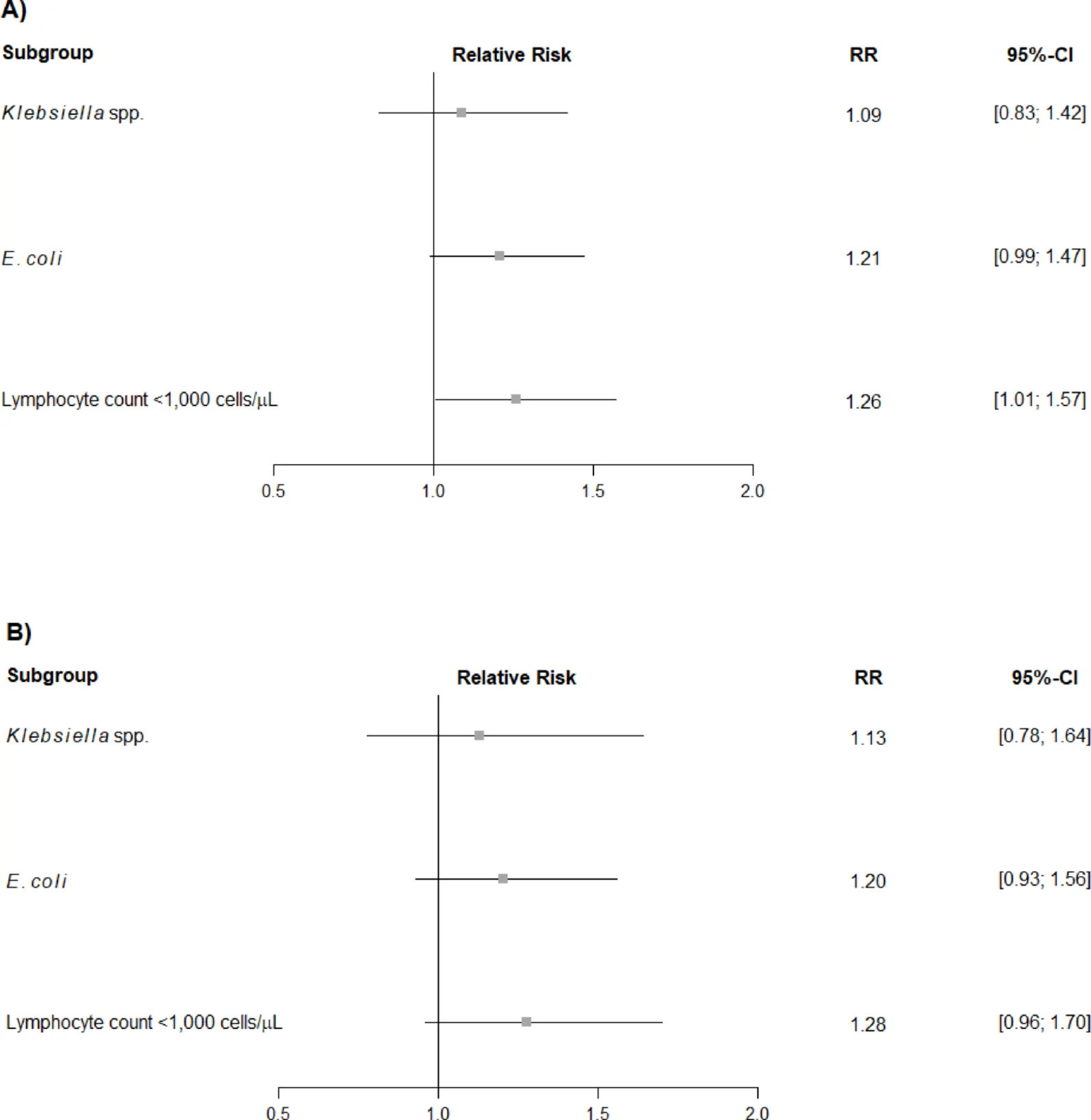
Forest plots of adjusted risk ratios for the association between TMP-SMX prophylaxis within the previous 30 days and resistance outcomes in selected subgroups of kidney transplant recipients with ESBL-E bloodstream infection. **A** TMP-SMX resistance. **B** TMP-SMX and ciprofloxacin co-resistance

Sensitivity analyses based on the multivariable models evaluated the association between recent TMP-SMX prophylaxis and resistance in selected clinical and microbiological subgroups (Fig. 1). For TMP-SMX resistance, point estimates suggested a higher probability of resistance in patients with *Klebsiella* spp. BSI (RR, 1.09; 95% CI 0.83–1.42), *E. coli* BSI (RR, 1.21; 95% CI 0.99–1.47), and lymphopenia (RR, 1.26; 95% CI 1.01–1.57), with statistical significance reached only in the lymphopenia subgroup (Fig. 1A).

For TMP-SMX and ciprofloxacin co-resistance, point estimates were also higher in patients with *Klebsiella* spp. BSI (RR, 1.13; 95% CI 0.78–1.64), *E. coli* BSI (RR, 1.20; 95% CI 0.93–1.56), and lymphopenia (RR, 1.28; 95% CI 0.96–1.70), although none of these associations reached statistical significance (Fig. 1B).

## Discussion

To our knowledge, this is the largest cohort of kidney transplant recipients with ESBL-E bloodstream infection reported to date. Our findings confirm that TMP-SMX resistance is highly prevalent in this population, with an overall rate of 82.7%. This high rate should be interpreted in the context of the study design, as all included patients had already developed ESBL-E BSI, a setting intrinsically enriched for multidrug resistance and therefore for resistance to non-beta-lactam agents such as TMP-SMX [15].

Recent TMP-SMX prophylaxis was associated with a higher probability of TMP-SMX resistance. Although the magnitude of the association was attenuated after IPTW and additional multivariable adjustment, prophylaxis remained associated with a 16% higher probability of resistance, with statistically significant results. These results should not be interpreted as demonstrating that prophylaxis causes resistance in the overall kidney transplant population. Rather, within this selected population, recent prophylaxis was associated with a greater probability that the causative isolate was resistant to TMP-SMX.

The exploratory CART analysis identified lymphopenia as the main splitting variable among patients receiving prophylaxis. Subsequent subgroup analyses showed that patients with both recent prophylaxis and lymphopenia had the highest observed prevalence of resistance (94.4%), and the association between prophylaxis and resistance was statistically significant within the lymphopenia subgroup. Therefore, lymphopenia should not be considered a confirmed effect modifier, but rather a marker of an exploratory subgroup in which TMP-SMX resistance was particularly frequent.

In transplant recipients, the absolute lymphocyte count is a pragmatic marker of the net state of immunosuppression, with clear implications for infectious risk [16] and has been repeatedly linked to late *Pneumocystis jirovecii* pneumonia, together with CMV infection, acute rejection, and exposure to lymphocyte-depleting therapies [12, 17–19]. Importantly, lymphopenia may also identify patients with greater cumulative or repeated TMP-SMX exposure than was captured by our binary measure of prophylaxis during the 30 days preceding BSI onset. In routine practice, prophylaxis is often prolonged or reintroduced in patients with delayed immune reconstitution, CMV infection, acute rejection, or exposure to lymphocyte-depleting therapies [17–20]. Accordingly, the observed pattern may be capturing a phenotype characterised by both deeper immunosuppression and greater cumulative or repeated exposure to TMP-SMX than was directly measured.

Chronic liver disease was independently associated with TMP-SMX resistance in the adjusted model, although this finding should be interpreted cautiously given the small number of patients with this condition and the absence of a clear mechanistic explanation.

We also found a high prevalence of co-resistance to TMP-SMX and ciprofloxacin, affecting more than 70% of the study population. Although co-resistance was more frequent among prophylaxis recipients in the unadjusted analysis, this association was attenuated and was no longer statistically significant after IPTW and multivariable adjustment. Nevertheless, the adjusted results remained directionally consistent with the significantly higher crude prevalence of co-resistance observed among prophylaxis recipients. The biological plausibility of co-resistance is supported by known mechanisms of co-selection: in Gram-negative bacilli, determinants of TMP-SMX resistance and plasmid-mediated quinolone resistance may co-localise within mobile genetic elements, favouring linked selection even when exposure is driven mainly by one of the two agents [21]. Thus, prolonged or repeated TMP-SMX exposure in highly immunosuppressed recipients could contribute not only to TMP-SMX resistance, but also to broader resistance phenotypes that compromise oral treatment options.

Exploratory sensitivity analyses also suggested that the association with recent prophylaxis was numerically more pronounced in *E. coli* than in *Klebsiella* spp. BSI for both resistance outcomes, possibly because prophylactic exposure may exert a more discernible selective effect in *E. coli*, whereas *Klebsiella* spp. may already have a higher baseline likelihood of multidrug resistance irrespective of recent TMP-SMX exposure.

We believe the clinical relevance of this result should be interpreted cautiously. These findings may be particularly relevant when empirical oral treatment is being considered in clinically stable kidney transplant recipients with suspected urinary-source infection, in whom TMP-SMX or fluoroquinolones might otherwise be considered before susceptibility results are available [22, 23]. In this context, recent TMP-SMX prophylaxis, particularly in patients with lymphopenia, may alert clinicians to a high probability that TMP-SMX will not be an available oral option. Treatment decisions should, however, remain based on isolate-specific susceptibility results.

Importantly, our findings should not be interpreted as a reason to withdraw TMP-SMX prophylaxis in kidney transplant recipients. TMP-SMX remains the standard preventive strategy against PJP and continues to offer important protection against opportunistic infections such as nocardiosis in solid organ transplant recipients [17, 24]. Instead, our results highlight a potential ecological trade-off and suggest that prophylaxis should be maintained according to guideline-recommended durations, but not unnecessarily prolonged in the absence of a clear clinical rationale [17, 20].

This study has several limitations. First, as a retrospective observational study, it is subject to residual confounding and potential selection biases. Restriction to patients with ESBL-E BSI may have introduced collider bias, and exclusion of patients who had received ≥ 2 days of active therapy before blood culture collection may have selected for more persistent or multidrug-resistant infections. Therefore, the findings should not be extrapolated to the overall kidney transplant population. Second, the exposure definition was necessarily limited, as prophylaxis was recorded as a binary variable within the previous 30 days and did not capture dose, schedule, cumulative duration, interruption, or recent discontinuation. Third, some potentially relevant variables, including prior ESBL colonisation, antecedent antibiotic exposure, and more granular markers of immunosuppressive burden, were not fully characterised. Fourth, the cohort covered the period from 2004 to 2016, during which clinical practices, resistance epidemiology, and susceptibility testing procedures may have changed. Finally, the CART and stratified analyses were exploratory, were performed in the same dataset, and were not externally validated.

In conclusion, among kidney transplant recipients with ESBL-E BSI, recent TMP-SMX prophylaxis was associated with a higher probability of TMP-SMX resistance after IPTW and multivariable adjustment. Exploratory CART and subgroup analyses identified patients with lymphopenia as a group with a particularly high prevalence of resistance. Clinically, this may be particularly relevant when empirical oral treatment is considered for suspected urinary-source infection in these patients and supports avoiding unjustified prolongation of prophylaxis beyond the duration recommended in clinical guidelines.

## Supplementary Information

Below is the link to the electronic supplementary material.

Supplementary Material 1

## Data Availability

The data that support the findings of this study are available from the corresponding author upon reasonable request. Additional Supporting Information may be found online in the Supporting Information tab for this article.

## Abbreviations

aRR: Adjusted risk ratio
BSI: Bloodstream infection
CART: Classification and Regression Tree
CI: Confidence interval
CMV: Cytomegalovirus
ESBL-E: Extended-spectrum beta-lactamase-producing Enterobacterales
IQR: Interquartile range
IPTW: Inverse probability treatment weightning
KT: Kidney transplantation
RR: Risk ratio
PJP: Pneumocystis jirovecii pneumonia
SOT: Solid organ transplant
TMP-SMX: Trimethoprim-sulfamethoxazole
