# Supplementary material for: Pneumocystis jirovecii prophylaxis with trimethoprim-sulfamethoxazole and resistance in ESBL-producing Enterobacterales bloodstream infections among kidney transplant recipients
Source: Antimicrob Resist Infect Control. 2026 Aug 18;15:142. doi: 10.1186/s13756-026-01799-4 (PMC13760918; doi:10.1186/s13756-026-01799-4)
Supplement: Supplementary file 1 [file 13756_2026_1799_MOESM1_ESM.docx]

**Supplementary Material**

**Table S1.** STROBE reporting checklist for cohort studies

| **Item** | **Reporting recommendation** | **Page(s)** |
| --- | --- | --- |
| 1. Title and abstract | Identify the study design and provide an informative and balanced summary of the methods and results. | 1, 6–7 |
| 2. Background/rationale | Explain the scientific background and rationale for the study. | 7–8 |
| 3. Objectives | State the specific objectives of the study. | 8 |
| 4. Study design | Present the main elements of the study design early in the manuscript. | 8 |
| 5. Setting | Describe the setting, participating centres, geographical scope, and relevant study dates. | 8–9 |
| 6. Participants | Describe eligibility criteria, participant selection, and exclusion criteria. | 8–9, 13 |
| 7. Variables | Clearly define the outcomes, exposure, potential confounders, effect modifiers, and diagnostic criteria. | 9–10; Supplementary Tables S2, S3, S5, and S6 |
| 8. Data sources and measurement | Describe the sources of data and the methods used to measure the main variables and microbiological outcomes. | 8–11 |
| 9. Bias | Describe the measures taken to address potential selection bias, confounding, centre effects, and other sources of bias. | 8, 12–14, 21–22 |
| 10. Study size | Explain how the study size was determined and report the number of included participants. | 8, 13 |
| 11. Quantitative variables | Explain how quantitative variables were handled and justify relevant thresholds or categories. | 10–12 |
| 12. Statistical methods | Describe statistical comparisons, adjustment for confounding, propensity-score weighting, missing-data handling, subgroup analyses, and sensitivity analyses. | 11–13; Figure 1; Supplementary Tables S2–S6 and Figures S1–S2 |
| 13. Participants in the analysis | Report the number of participants included and describe relevant exclusions. | 8–9, 13 |
| 14. Descriptive data | Report participant characteristics, exposures, potential confounders, and the extent of missing data. | 13–14; Table 1, 30–31 |
| 15. Outcome data | Report the frequency of TMP-SMX resistance and TMP-SMX/ciprofloxacin co-resistance. | 14–18; Tables 1–2; 30-32; Supplementary Tables S3 and S6 |
| 16. Main results | Report unadjusted and adjusted estimates with 95% confidence intervals and identify the covariates included in adjusted analyses. | 14–18; Table 3; Supplementary Tables S2, S4, and S5 |
| 17. Other analyses | Report exploratory CART, interaction, stratified, subgroup, and sensitivity analyses. | Pages 14–18; Figure 1; Supplementary Tables S3 and S6; Figures S1 and S2 |
| 18. Key results | Summarise the principal findings in relation to the study objectives. | 18–20 |
| 19. Limitations | Discuss study limitations and the probable direction or magnitude of potential biases. | 21–22 |
| 20. Interpretation | Provide an appropriately cautious interpretation considering the objectives, limitations, multiplicity of analyses, and available evidence. | 18–22 |
| 21. Generalisability | Discuss the generalisability of the findings and the selected nature of the study population. | 18-19, 21–22 |
| 22. Funding | State the sources of funding and the role of the funders. | 23–24 |

**Table S2.** IPTW-weighted univariable and multivariable modified Poisson regression analyses of factors associated with trimethoprim-sulfamethoxazole resistance in kidney transplant recipients with bloodstream infections caused by extended-spectrum β-lactamase-producing Enterobacterales^1^

| **Variable** | **Univariable analysis** | | | **Multivariable analysis** | | | |
| --- | --- | --- | --- | --- | --- | --- | --- |
|  | **RR** | **95% CI** | **P value** | **aRR** | **95% CI** | **P value** |  |
| **Male gender** | 1.06 | 0.92–1.22 | 0.40 |  |  |  |  |
| **Age, per 1-year increase** | 1.00 | 0.99–1.00 | 0.22 |  |  |  |  |
| **Comorbidities** |  |  |  |  |  |  |  |
| Diabetes mellitus | 1.00 | 0.88–1.13 | 0.99 |  |  |  |  |
| Solid tumour | 0.94 | 0.84- 1.05 | 0.26 |  |  |  |  |
| Chronic kidney disease | 0.98 | 0.88–1.09 | 0.72 |  |  |  |  |
| Chronic obstructive pulmonary disease | 0.94 | 0.79–1.12 | 0.50 |  |  |  |  |
| Chronic liver disease | 1.18 | 1.05–1.34 | 0.008 | 1.18 | 1.04–1.34 | 0.01 |  |
| Chronic heart failure | 0.89 | 0.39–1.15 | 0.36 |  |  |  |  |
| Cerebrovascular disease | 1.04 | 0.91–1.19 | 0.59 |  |  |  |  |
| Charlson comorbidity index, per 1-point increase | 0.99 | 0.96–1.01 | 0.23 |  |  |  |  |
| McCabe rapidly or ultimately fatal | 1.04 | 0.89–1.21 | 0.63 |  |  |  |  |
| **Maintenance immunosuppressive therapy** |  |  |  |  |  |  |  |
| *Mycophenolic acid/mycophenolate mofetil* | 0.92 | 0.81–1.04 | 0.18 |  |  |  |  |
| *Tacrolimus* | 1.12 | 0.88–1.43 | 0.35 |  |  |  |  |
| *Sirolimus* | 1.23 | 0.95-1.59 | 0.11 |  |  |  |  |
| *Everolimus* | 1.12 | 0.92–1.37 | 0.26 |  |  |  |  |
| *Ciclosporin* | 0.87 | 0.62–1.21 | 0.39 |  |  |  |  |
| *Azathioprine* | 1.12 | 0.92–1.36 | 0.26 |  |  |  |  |
| *Corticosteroids* | 0.95 | 0.80-1.13 | 0.54 |  |  |  |  |
| ***Induction immunosuppressive therapy*** |  |  |  |  |  |  |  |
| *Basiliximab* | 0.86 | 0.75–0.99 | 0.04 |  |  |  |  |
| *Antithymocyte globulin* | 1.06 | 0.94–1.20 | 0.31 |  |  |  |  |
| **Acute graft rejection within the previous 30 days** | 1.04 | 0.84–1.28 | 0.72 |  |  |  |  |
| **TMP-SMX prophylaxis within the previous 30 days** | 1.18 | 1.02–1.37 | 0.03 | 1.16 | 1.00–1.35 | 0.05 |  |
| **Lymphocyte count <1,000 cells/µL** | 1.13 | 1.00–1.28 | 0.05 | 1.09 | 0.96–1.23 | 0.19 |  |
| **CMV replication within the previous 30 days** | 1.16 | 1.03–1.30 | 0.01 |  |  |  |  |
| **CMV disease within the previous 30 days** | 1.25 | 1.15–1.35 | <0.001 |  |  |  |  |
| **Intensive care unit stay before BSI onset** | 0.96 | 0.75-1.22 | 0.71 |  |  |  |  |
| **Mechanical ventilation before BSI onset** | 0.96 | 0.75-1.22 | 0.71 |  |  |  |  |
| **Time from kidney transplantation to BSI ≤90 days** | 1.08 | 0.94–1.25 | 0.26 |  |  |  |  |
| **Aetiology, reference: *Escherichia coli*** |  |  |  |  |  |  |  |
| *Klebsiella pneumoniae* | 1.18 | 0.97–1.42 | 0.09 | 1.10 | 0.94–1.36 | 0.18 |  |
| Other pathogens | 1.00 | 0.65–1.53 | 1.00 |  |  |  |  |
| **Urinary tract source** | 1.19 | 0.89–1.62 | 0.25 |  |  |  |  |

^1^Risk ratios were estimated using modified Poisson regression with a log link and robust variance. Analyses were weighted using stabilised inverse probability of treatment weights truncated at the 2.5th and 97.5th percentiles. Centre effects were addressed through centre-clustered robust variance.

Abbreviations: aRR, adjusted risk ratio; BSI, bloodstream infection; CI, confidence interval; CMV, cytomegalovirus; IPTW, inverse probability of treatment weighting; NA, not applicable; NE, not estimated; RR, risk ratio; TMP-SMX, trimethoprim-sulfamethoxazole.

**Table S3.** Observed TMP-SMX resistance and exploratory IPTW-adjusted effect-modification analysis according to lymphopenia status^1^

| **Lymphopenia status** | **TMP-SMX prophylaxis** | **Observed TMP-SMX resistance, n/N (%)** | **IPTW-adjusted RR** | **95% CI** | **P value** |
| --- | --- | --- | --- | --- | --- |
| No lymphopenia | No | 48/65 (73.8) | Reference | — | — |
|  | Yes | 27/34 (79.4) | 0.97 | 0.68–1.37 | 0.840 |
| Lymphopenia <1,000 cells/µL | No | 73/98 (74.5) | Reference | — | — |
|  | Yes | 119/126 (94.4) | 1.23 | 1.00–1.51 | 0.048 |

^1^	Observed resistance frequencies are presented as unweighted counts and percentages. Adjusted risk ratios were estimated from the IPTW-weighted modified Poisson model with centre-clustered robust variance.

**Table S4.** Risk ratios for TMP-SMX and ciprofloxacin co-resistance according to prior TMP-SMX prophylaxis

| **Model** | **RR for TMP-SMX prophylaxis** | **95% CI** | **P value** |
| --- | --- | --- | --- |
| Unadjusted | 1.16 | 1.01–1.34 | 0.04 |
| IPTW with centre-clustered robust variance | 1.14 | 0.99–1.31 | 0.07 |
| IPTW with centre-clustered robust variance and additional adjustment for lymphopenia, chronic liver disease, and aetiology* | 1.11 | 0.97–1.28 | 0.10 |

*See Supplementary Table S5. Abbreviations: CI, confidence interval; IPTW, inverse probability of treatment weighting; RR, risk ratio; TMP-SMX, trimethoprim-sulfamethoxazole.

**Table S5**. IPTW-weighted univariable and multivariable modified Poisson regression analyses of factors associated with trimethoprim-sulfamethoxazole and ciprofloxacin co-resistance in kidney transplant recipients with bloodstream infections caused by extended-spectrum β-lactamase-producing Enterobacterales^1^

| **Variable** | **Univariable analysis** | | | **Multivariable analysis** | | | |
| --- | --- | --- | --- | --- | --- | --- | --- |
|  | **RR** | **95% CI** | **P value** | **aRR** | **95% CI** | **P value** |  |
| **Male gender** | 1.12 | 0.93–1.34 | 0.23 |  |  |  |  |
| **Age, per 1-year increase** | 1.00 | 0.99–1.00 | 0.73 |  |  |  |  |
| **Comorbidities** |  |  |  |  |  |  |  |
| Diabetes mellitus | 1.00 | 0.85–1.17 | 0.96 |  |  |  |  |
| Solid tumour | 0.93 | 0.79- 1.10 | 0.40 |  |  |  |  |
| Chronic kidney disease | 0.99 | 0.91–1.07 | 0.74 |  |  |  |  |
| Chronic obstructive pulmonary disease | 1.04 | 0.89–1.23 | 0.60 |  |  |  |  |
| Chronic liver disease | 1.19 | 1.01–1.40 | 0.04 | 1.17 | 0.99–1.38 | 0.06 |  |
| Chronic heart failure | 0.95 | 0.72–1.27 | 0.73 |  |  |  |  |
| Cerebrovascular disease | 1.14 | 0.94–1.37 | 0.19 |  |  |  |  |
| Charlson comorbidity index, per 1-point increase | 0.98 | 0.95–1.01 | 0.17 |  |  |  |  |
| McCabe rapidly or ultimately fatal | 0.97 | 0.76–1.24 | 0.82 |  |  |  |  |
| **Maintenance immunosuppressive therapy** |  |  |  |  |  |  |  |
| *Mycophenolic acid/mycophenolate mofetil* | 1.06 | 0.78–1.44 | 0.70 |  |  |  |  |
| *Tacrolimus* | 1.15 | 0.85–1.57 | 0.35 |  |  |  |  |
| *Sirolimus* | 1.39 | 0.89–2.18 | 0.15 |  |  |  |  |
| *Everolimus* | 0.82 | 0.47–1.44 | 0.47 |  |  |  |  |
| *Ciclosporin* | 0.86 | 0.59–1.25 | 0.41 |  |  |  |  |
| *Azathioprine* | 1.18 | 0.91–1.52 | 0.21 |  |  |  |  |
| *Corticosteroids* | 0.85 | 0.66-1.08 | 0.17 |  |  |  |  |
| ***Induction immunosuppressive therapy*** |  |  |  |  |  |  |  |
| *Basiliximab* | 0.83 | 0.68–1.01 | 0.06 |  |  |  |  |
| *Antithymocyte globulin* | 1.10 | 0.92–1.31 | 0.28 |  |  |  |  |
| **Acute graft rejection within the previous 30 days** | 0.93 | 0.67–1.29 | 0.65 |  |  |  |  |
| **TMP-SMX prophylaxis within the previous 30 days** | 1.14 | 0.99–1.31 | 0.07 | 1.11 | 0.97–1.28 | 0.10 |  |
| **Lymphocyte count <1,000 cells/µL** | 1.11 | 0.98–1.25 | 0.09 | 1.08 | 0.96–1.21 | 0.20 |  |
| **CMV replication within the previous 30 days** | 1.24 | 1.03–1.50 | 0.03 |  |  |  |  |
| **CMV disease within the previous 30 days** | 1.30 | 1.08–1.56 | 0.007 |  |  |  |  |
| **Intensive care unit stay before BSI onset** | 0.96 | 0.71-1.29 | 0.77 |  |  |  |  |
| **Mechanical ventilation before BSI onset** | 1.06 | 0.93-1.20 | 0.42 |  |  |  |  |
| **Time from kidney transplantation to BSI ≤90 days** | 1.03 | 0.88–1.19 | 0.75 |  |  |  |  |
| **Aetiology, reference: *Escherichia coli*** |  |  |  |  |  |  |  |
| *Klebsiella pneumoniae* | 1.12 | 0.97–1.30 | 0.11 | 1.09 | 0.98–1.22 | 0.12 |  |
| Other pathogens | 1.02 | 0.22-4.80 | 0.98 |  |  |  |  |
| **Urinary tract source** | 1.24 | 0.89–1.72 | 0.20 |  |  |  |  |

^1^Risk ratios were estimated using modified Poisson regression with a log link and robust variance. Analyses were weighted using stabilised inverse probability of treatment weights truncated at the 2.5th and 97.5th percentiles. Centre effects were addressed through centre-clustered robust variance.

Abbreviations: aRR, adjusted risk ratio; BSI, bloodstream infection; CI, confidence interval; CMV, cytomegalovirus; IPTW, inverse probability of treatment weighting; NA, not applicable; NE, not estimated; RR, risk ratio; TMP-SMX, trimethoprim-sulfamethoxazole.

**Table S6.** Observed TMP-SMX and ciprofloxacin co-resistance and exploratory IPTW-adjusted effect-modification analysis according to lymphopenia status^1^

| **Lymphopenia status** | **TMP-SMX prophylaxis** | **Observed TMP-SMX and ciprofloxacin co-resistance, n/N (%)** | **IPTW-adjusted RR** | **95% CI** | **P value** |
| --- | --- | --- | --- | --- | --- |
| No lymphopenia | No | 43/65 (66.2) | Reference | — | — |
|  | Yes | 21/34 (61.8) | 0.99 | 0.67–1.46 | 0.96 |
| Lymphopenia <1,000 cells/µL | No | 63/98 (64.3) | Reference | — | — |
|  | Yes | 100/126 (79.4) | 1.28 | 0.96–1.70 | 0.08 |

^1^	Observed resistance frequencies are presented as unweighted counts and percentages. Adjusted risk ratios were estimated from the IPTW-weighted modified Poisson model with centre-clustered robust variance.

**Figure S1.** CART model identifying variables associated with resistance to trimethoprim-sulfamethoxazole.

Note: Exploratory CART model developed in the complete cohort of 323 patients. Twenty-three candidate predictors were entered; the tree with the lowest relative cost retained three splitting variables and comprised four terminal nodes. No independent test or holdout sample was used.

**Figure S2. CART model identifying variables associated with co-resistance to trimethoprim-sulfamethoxazole and ciprofloxacin.**

Note: Exploratory CART model developed in the complete cohort of 323 patients. Twenty-three candidate predictors were entered; the tree with the lowest relative cost retained three splitting variables and comprised four terminal nodes. No independent test or holdout sample was used.
